# Prevalence and molecular characterization of some circulating strains of the *peste-des-petits*-ruminants virus in Saudi Arabia between 2014–2016

**DOI:** 10.7717/peerj.9035

**Published:** 2020-05-27

**Authors:** Maged Gomaa Hemida, Hussain Mohammed Alghadeer, Mohammed Alhammadi, Sayed Ali

**Affiliations:** 1 Department of Microbiology, College of Veterinary Medicine, King Faisal University, Al-Hufuf, Al-Hasa, Saudi Arabia; 2 Department of Virology, Faculty of Veterinary Medicine, Kafrelsheikh University, Kafrelsheikh, Kafrelsheikh, Egypt; 3 Department of Virology, Central Veterinary Diagnostic Laboratory, Minstry of Water, Enviroment and Agriculture, Riyadh, Riyadh, Saudi Arabia; 4 Department of Family and Community Medicine, College of Medicine, King Faisal University, Al-Hufuf, Al-Hasa, Saudi Arabia

**Keywords:** SRMV, ELISA, PCR, Real Time PCR, Small ruminants, lineage IV, Saudi Arabia

## Abstract

**Background:**

The peste-des-petits-ruminants virus (PPRV) is a highly devastating virus of small ruminants in many parts of the world, including the Kingdome of Saudi Arabia. Therefore, our objectives were (1) to conduct a molecular prevalence study of PPRV in sheep and goat across the KSA and (2) to isolate and identify currently circulating PPRV lineages. Swabs and tissue specimens were collected from 97 herds suspected to be infected with PPRV across the Kingdome of Saudi Arabia (KSA). Testing for the presence of the virus was done by the real-time PCR. Confirmation of the identity of the reactions was done by the gel-based-PCR then by sequencing of the partial PPRV genome.

**Results:**

Our results revealed that 24.1% of the tested specimens were PPRV-positive. Isolation of PPRV was successful from samples using the Vero cell line. Sequence analysis of some partial PPRV genes (N, F, M, L, P, and H) revealed that these strains were belonging to lineage IV of the PPRV.

**Conclusions:**

This is the first study to conduct both the nationwide prevalence, isolation, and molecular characterizations of the PPRV in the KSA. Continuous surveillance and monitoring of the circulating strains of PPRV among sheep and goats will contribute substantially to the global eradication campaign of such a virus.

## Introduction

PPRV is considered one of the significant concerns of small ruminant animal production. PPRV is endemic in many regions of the world, especially the Middle East, Africa, India, and China. PPRV infection results in high morbidity and mortality rates, which can be as high as 100% (Abubakr, Ali & Khan, 2008). There is an ongoing collaboration between the OIE and the FAO to contain the PPRV throughout the world, especially in the endemic regions at 2030 (Food Agriculture Organization (FAO), 2014). Although PPRV was identified a long time ago (Hamdy et al., 1976; Lefèvre & Diallo, 1990; Whitney, Scott & Hill, 1967), several outbreaks are still occurring in many of the indicated regions (Albina et al., 2013).

The PPRV is a causative agent of the *Pest des petite* ruminants (PPR). It is a significant viral disease affecting small ruminants, especially in Africa and Asia (Alemu et al., 2019; Mohmoud et al., 2018). Infected animals are capable of shedding the virus in their excretions. Therefore, the spread of the virus between animals in close contact is relatively straightforward, as transmission via direct contact is efficient (Radostits et al., 2009). Direct contact between PPRV-infected and naïve animals facilitates the transmission through both respiratory and fecal-oral routes (Dhar et al., 2002; Muthuchelvan et al., 2006). The PPRV infection is associated with a wide range of clinical signs, including high fever, nasal and lachrymal discharges, diarrhea, and pneumonia. Mortality due to severe PPRV infection is widespread in small ruminants (Dhar et al., 2002; Muthuchelvan et al., 2006). The PPRV belongs to the family *Paramyxoviridae*, the genus *Morbillivirus*, species *Small ruminant morbillivirus* (Amarasinghe et al., 2018). The viral genome is a linear, none-segmented, negative-sense single-stranded RNA that is about 15,948 nucleotides in length (Bailey et al., 2005; Norrby & Oxman, 1990). It encodes eight proteins. Six of these proteins are structural: the Fusion (F), Nucleocapsid (N), Phosphoprotein (P), Large (L), Haemagglutinin (H) and Matrix (M) proteins (Bailey et al., 2005; Baron & Barrett, 1995; Barrett, 2001; Diallo, 1990; Norrby & Oxman, 1990). PPRV was first reported in Africa on the Ivory Coast (Gargadennec & Lalanne, 1942). However, it has now become of particular concern due to the number of outbreaks that have been reported in many parts of the world, especially Asia and Africa (El-Yuguda et al., 2009; Nanda et al., 1996; Obi et al., 1983; Ozkul et al., 2002; Shaila et al., 1996). Only one PPRV serotype has been identified to date, and four lineages (I–IV) of this serotype have been reported (Luka et al., 2011).

Lineage IV has originally emerged in the Middle East and some other Asian countries (Kwiatek et al., 2011; Parida et al., 2015). However, lineage IV was recently reported in many endemic regions I Africa suggesting there are two distinct groups of this linage circulating in both Asia and Africa (Parida et al., 2015). In the Arabian Peninsula, the occurrence of two PPRV lineages was confirmed. Lineage III has been identified in small ruminant flocks in Oman (Hedger, Barnett & Gray, 1980; Taylor, Al-Busaidy & Barrett, 1990) and the UAE (Furley, Taylor & Obi, 1987; Muniraju et al., 2014). However, lineage IV has been identified in the KSA based on the partial sequence of the PPRV-N and F genes (Asmar et al., 1980; Banyard et al., 2010). Several studies have conducted serosurveillance of PPRV across the KSA (AL-Afaleq et al., 2004; Ahmed, Muaz & Luai, 2016 Al-Dubaib, 2008; Boshra et al., 2015). Furthermore, a seroprevalence study of PPRV infection was conducted in small ruminants during the early 1980s (Asmar et al., 1980). Additionally, PPRV outbreaks occurred in sheep and goats in the Al-Hasa province of the KSA in the early nineties, as well as in 2000 (Abu Elzein et al., 1990; Al Naeem, Elzein & Al-Afaleq, 2000). The 2002 PPRV outbreak was shown to result in 100% mortality among the affected animals (Housawi et al., 2004). Another large-scale seroprevalence study was also conducted on targeted animals from 11 different locations across the central region of the KSA. The study reported anti-PPRV antibody prevalence rates of 36.59% and 55.09% among the tested sheep and goat populations, respectively (Al-Dubaib, 2008). More recently, a new study conducted a seroprevalence in the three main regions across the countries of Al-Hasa, Riyadh, and Assir. We found that 40%, 85%, and 80% of the tested animals were seropositive for PPRV antibodies in each of the regions, respectively (Boshra et al., 2015). Recently, detection of PPRV in a group of wild ruminants such as Arabian gazelles was reported by other colleagues (Sharawi et al., 2010). PPRV has been monitored in the KSA for more than three decades; however, despite active vaccination campaigns with a live vaccine made with an attenuated Nigerian isolate of the virus (Nigeria 75/1), many PPR outbreaks are still being reported (El-Yuguda et al., 2009). The main objectives of the current study were (1) to perform a nationwide investigation of the molecular prevalence of PPRV across the KSA, (2) to isolate the currently circulating PPRV strains in small ruminants raised in the KSA, (3) to decode the full-length genome of the currently circulating PPRV strains in the country and (4) to conduct a molecular characterization of the isolated PPRV strains.

## Materials & Methods

### Vertebrate animal study

This project was approved by the King Faisal University deanship of scientific research animal ethics committee) and approval reference number (KFU-DSR-project No: 150074).

### Study population

**Figure 1:**
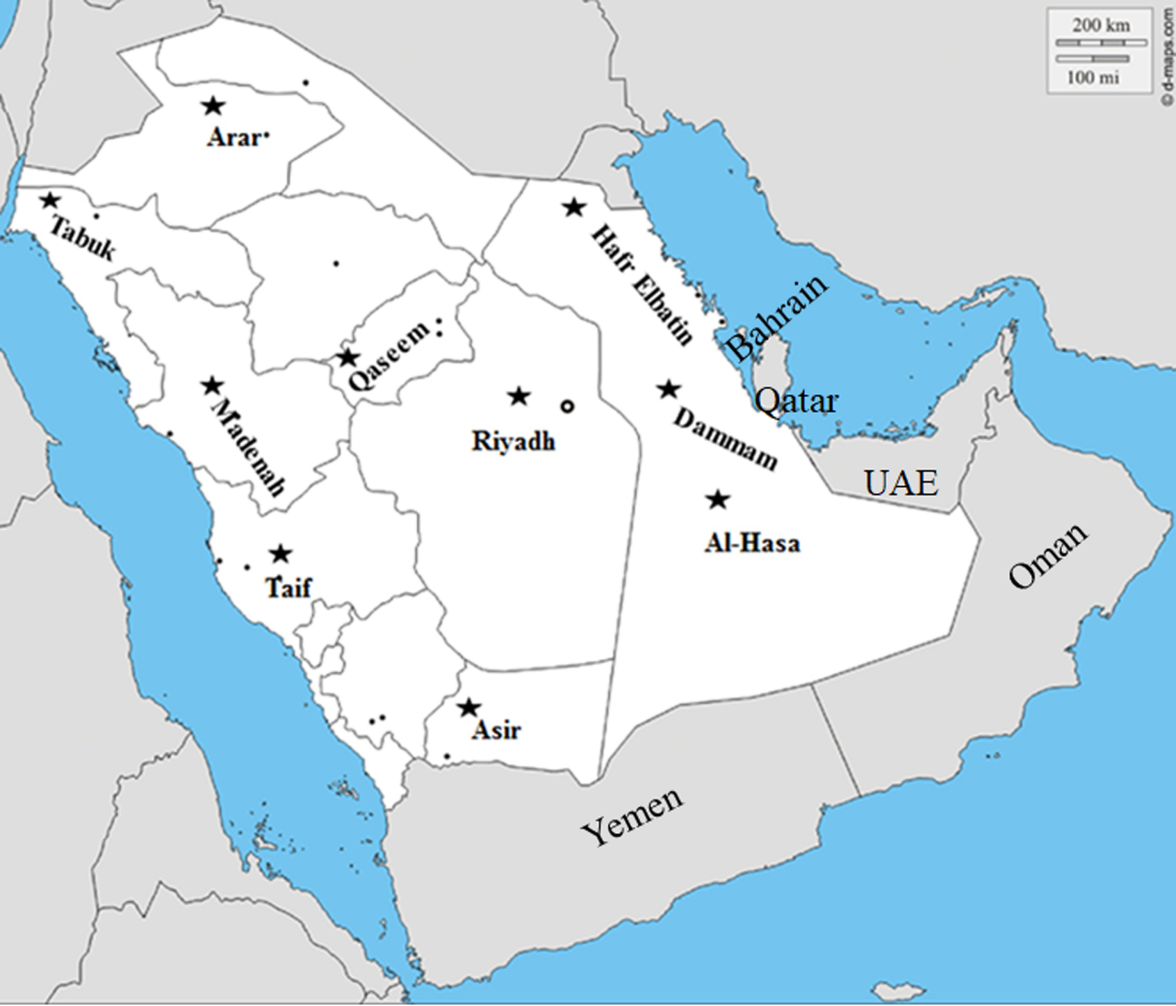
Geographical distribution of PPRV suspected outbreaks. Map showing the geographical distribution of the suspected PPRV outbreaks in small ruminants across Saudi Arabia from 2014–2016 (http://dmap.com/, 2019).

A surveillance campaign was conducted to assess the current situation of PPRV infection among small ruminants in KSA during 2014–2016. A total of 97-PPRV outbreaks, including 75 in sheep and 22 in goats, were observed during the duration of our surveillance (Table 1). The average size of these herds is 10–20 animals. We used the following criteria for animal sampling. First, we tried to collect specimens from at least 10% per each of the investigated flocks. However, the number of sampled animals was primarily affected by the consent from owners. Second, we selected the most severely affected animals that showed clinical signs of infection, specifically, respiratory, enteric, and ocular manifestations. We applied the purposeful sampling approach, as previously described (Patton, 2002). Specimens from 223 animals from confirmed PPRV outbreaks (including oral, nasal and lachrymal swabs, as well as tissues including lung, liver, spleen, lymph nodes and intestines) were collected from flocks of small ruminants across the KSA between Jan 2014 and Oct 2016 (Fig. 1). We also sampled some outwardly appearing healthy animals.

**Table 1:** Summary of the SRMV outbreaks in small ruminants across Saudi Arabia between Jan 2014–Oct 2016 and their geographical distribution 2014–2016.

**N**	**City**	**2014**			**chi square**	*p* value	**2015**			**chi square**	*p* value	**2016**			**chi square**	*p* value	**Total**			**chi square**	P^*^ value
		No	Sh	Gt			No	Sh	Gt			No	Sh	Gt			No	Sh	Gt		
1	**Ryd**	24	20	4	10.66	0.001	17	14	3	7.11	0.01	8	3	5	0.5	NS	49	37	12	12.75	0.001
2	**Qas**	1	1	0	1	**NS**	0	0	0	NA	NA	0	0	0	NA	NA	1	1	0	1	NS
3	**Dmm**	1	1	0	1	**NS**	2	2	0	2	**NS**	0	0	0	NA	NA	3	3	0	3	NS
4	**Hasa**	0	0	0	NA	NA	5	5	0	5	0.05	0	0	0	NA	NA	5	5	0	5	0.05
5	**HBT**	1	1	0	1	**NS**	0	0	0	NA	NA	0	0	0	NA	NA	1	1	0	1	NS
6	**Mad**	5	5	0	5	0.05	1	1	0	1	**NS**	0	0	0	NA	NA	6	6	0	6	0.05
7	**Asir**	9	6	3	1	**NS**	9	6	3	1	**NS**	2	1	1	0	**NS**	20	13	7	1.8	NS
8	**Taif**	3	1	2	0.33	**NS**	6	5	1	2.66	**NS**	1	1	0	1	**NS**	10	7	3	1.6	NS
9	**TBK**	0	0	0	NA	NA	0	0	0	NA	NA	1	1	0	1	**NS**	1	1	0	1	NS
10	**Arar**	0	0	0	NA	NA	0	0	0	NA	NA	1	1	0	1	**NS**	1	1	0	1	NS
	Total	44	35	9	15.36	0.001	40	33	7	17	0.01	13	7	6	0.07	**NS**	97	75	22	28.5	0.001

**Notes.**

Ryd Riyadh Qas Qaseem Dmm Dammam Hasa Al-Hasa Mad Madinah TBK Tabuk NS Non-significant NA Not Applicable Sh sheep Gt Goat

* The *P*-value indicates there is a significant difference between the total numbers of PPRV outbreaks in sheep and goats independently in various regions as well as per each year across the KSA during the tenure of this study (2014–2016).

### Tissue specimens

Tissue specimens were collected from selected animals that were showing typical clinical signs of PPRV during the necropsy examination during each outbreak. We collected ten grams from each organ that showed gross pathological changes (lung, liver, spleen, intestine, and some regional lymph nodes,especially the mesenteric and retropharyngeal). Samples were collected under complete aseptic conditions. We prepared 10% tissue suspensions for each tissue, as previously described, with some modifications (Troung et al., 2014). Briefly, one gram of tissue specimen was mixed with 9 ml of PBS. Sterile sand was added, and the tissue was triturated using a sterile mortar and pestle. The tissue suspensions obtained were centrifuged for 10 min at 4 °C in a cooled centrifuge. We then collected the tissue supernatants and stored them at −80 °C for further use.

### Swabs

A total of 23 swabs (oral, nasal, and lachrymal) were collected from several suspected PPRV outbreaks. Swabs were obtained as previously described, with some modifications (Troung et al., 2014). The collected swabs were transferred to a viral transport medium containing Dulbecco’s Modified Eagle Medium (DMEM; Gibco™) that included 10% fetal bovine serum (FBS; Invitrogen, Carlsbad, CA) and an antibiotic cocktail (penicillin, 10,000 units/mL, and streptomycin, 10,000 g/mL). Swabs were vortexed and centrifuged at 5000 RPM for 10 min at 4 °C. The supernatants were collected and stored at (−80 °C) for further testing.

### RNA extraction and reverse transcription

Total viral RNA was extracted from both swabs and tissue specimens using a viral RNA extraction kit (RNeasy Mini Kit, QIAGEN, cat. nos. 74104 and 74106). RNA extraction was performed according to the manufacturer’s instructions, as previously described elsewhere (Kumar et al., 2014). The eluted viral RNAs were stored at −80 °C for further testing.

### Detection of PPRV by TaqMan real-time RT-PCR assay (qPCR)

The qPCR amplification of the PPRV genome was carried out by using a *Pestes des Petits Ruminants* (PPRV) ©2013 Genekam Biotechnology AG, Ref; K229, Germany). Amplification was performed using qPCR (Light cycler 2.0, Serial No: 1416238, Roche, Switzerland). Each reaction of 20 µl contained 2.4 µl of Mg CL solution, 2 µl of Roche Master (LightCycler Fast Start DNA Master HybProbe Roche, Germany, Cat. No. 03 003 248 001), 2 µl of reagent mix (specific reagent containing oligonucleotides and probes), 2 µl of IC mix (reagent containing oligonucleotides, probes, and cDNAs), and 6.6 µl of PCR-grade water. A 15-µl aliquot of each reaction mixture was added to light cycler capillaries. Finally, 5 µl of the cDNA was added to the light cycler capillaries. The kit’s amplification conditions were applied to test for the presence of the PPRV genome in the samples.

We used one set of oligonucleotides that amplify the partial PPRV-F gene as previously described elsewhere (Ozkul et al., 2002) (the PPRV-F, 5′-AGTACAAAAGATTGCTGAT- CACAGT-3′, and the PPRV-R, 5′-GGGTCTCGAAGGCTAGGCCCGAATA-3′).

### Detection of PPRV by classical PCR assay

A conventional PCR assay was used to amplify the partial PPRV-F gene using the oligonucleotides as previously described (Ozkul et al., 2002); these oligonucleotides are listed above. The first-strand (cDNA) synthesis was performed according to the protocol of the kit (Roche, cat. No. 04 379 012 001). The PCR amplification conditions were as follows: denaturation (1 min at 95 °C), annealing (1 min at 60 °C), and extension (1 min at 72° C). These steps were repeated for 40 cycles, followed by a final extension at 72  °C for 10 min. We used both negative (non-template DNA), and positive (Nigeria 75/1strain) controls alongside each reaction. The RT-PCR products were resolved in 1% agarose gels containing SYBR^®^ Safe DNA Gel Stain (Life Technologies). The amplified DNA fragments were visualized under ultraviolet light, and photographs were captured with a gel documentation system (Bio-Rad Laboratories, Inc., Hercules, California, USA). The target amplified partial PPRV-F gene bands were excised from the gel and purified using a QIAquick Gel Extraction Kit (Cat No/ID: 28704) according to the protocol of the kit manufacturer. The purified reactions were eluted in a 50-µl elution buffer.

### Sanger method of sequencing and sequencing analysis

We selected some RT-PCR-positive specimens that were collected from animals in Hafar Al-Batin (a region located along the border of Saudi Arabia and other Gulf countries such as Kuwait). We sequenced these specimens by the Sanger method to confirm the identity of the amplicons. The purified PCR products were sequenced in both directions using the original oligonucleotides used in the PCR amplification. We assembled the obtained sequences into one contig and performed a nucleotide BLAST through NCBI (https://blast.ncbi.nlm.nih.gov/Blast.cgi?CMD=Web&PAGE_TYPE=BlastHome). The obtained sequences were aligned and compared with the PPRV control vaccine strain, as well as the other PPRV genome sequences available in GenBank.

### Next-generation sequencing (NGS)

To obtain the full-length genome sequence of the PPRV, we used another positive PPRV specimen from sheep lung tissue that was isolated from Riyadh and sequenced it by the NGS approach described below. Briefly, the short ds-cDNA fragments were joined by specific adaptor sequences. The desired amplicons were obtained using agarose gel electrophoresis. Then, qPCR amplification of the obtained RNA libraries was conducted. Quantification of these libraries was performed by the qPCR. Reactions were performed according to the kit manufacturers’ instructions (San Diego, CA, USA). Further qualification was conducted using a Bioanalyzer (Agilent2100). The cDNA library was sequenced using the HiSeqTM 2000 platform (Illumina; Macrogen Corp., Maryland, USA). The quality of the sequences was checked by the FastQC (v0.10.0) program (http://www.bioinformatics.babraham.ac.uk/projects/fastqc/). Data analysis was conducted by filtering the raw sequences and removing scrambled sequences using Trimmomatic software (Bolger, Lohse & Usadel, 2014). Trimmed reads were then assembled using the Trinity program, as previously described (Grabherr et al., 2011). All obtained sequences were assembled into one contig, and the expression level of each contig was determined by the fragments/ kilobase. For annotation, the consensus sequences were searched against the NCBI-Nucleotide (NT) database using blastn (2.2.26+version).

### PPRV isolation

PPRV was isolated using Vero cell lines (ATCC, CCL-81), as previously described (Hegde et al, 2009). We selected ten samples from seven laboratory-confirmed animals for the purpose of PPRV isolation. These samples included six tissue suspensions (lung, liver, spleen) and four swabs (oral and lachrymal). In some cases, we used swabs and one tissue from one animal. The rationale behind this was the presence of some pathological changes in this organ suggestive for the PPRV infection. These specimens were selected based on their low Ct values during the qPCR testing, which represented the high RNA concentration in these specimens. We used 100 µl of the tested samples (tissue suspension or swab) to inoculate 90% confluent cells. After one hour, the virus inoculum was removed, and cells were washed three times with PBS. This was followed by adding fresh DMEM maintenance media that contained fetal bovine serum (FBS; Invitrogen, Carlsbad, CA). The inoculated cells were incubated at 37 °C and 5% CO_2._ Cells were monitored on a daily basis under a light microscope for the development of cytopathic effects (CPE) for up to 7 days post-infection. The PPRV vaccine (Nigeria 75/1) strain was obtained from the Veterinary Vaccines Production Center in Riyadh, KSA. The lyophilized vaccine was diluted in 1 ml of DMEM, and 100 µl of the diluted vaccine was inoculated into the Vero cell culture flasks and monitored as described above.

**Figure 2:**
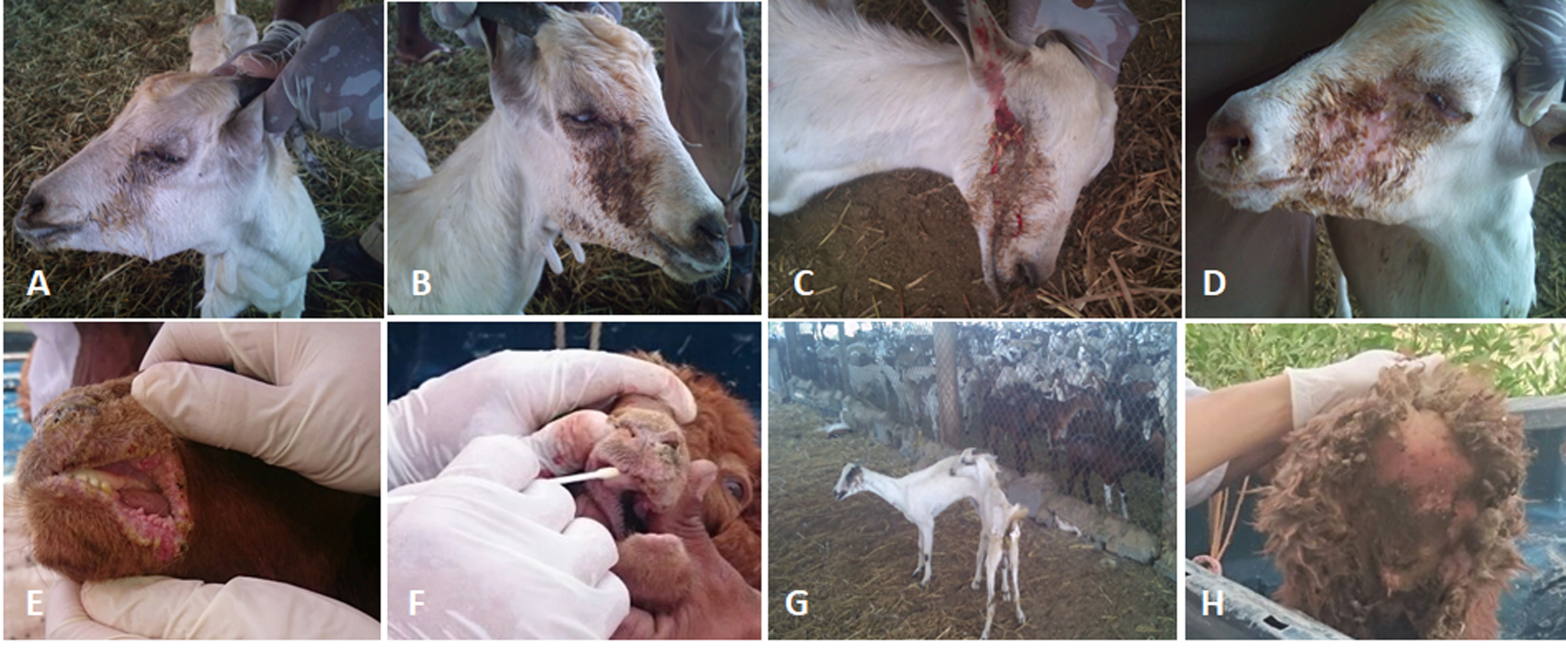
The clinical pictures of PPRV infections in native small ruminants. The clinical examination of some PCR positives PPRV infected local small ruminants breeds. (A) Sheep showing mucopurulent discharge from the nostrils; (B) sheep showing erosion in the buccal cavity around the corners of the mouth; (C) goat showing excessive lachrymal discharge; (D) goat showing complete blindness and closure of the eyelid; (E) sheep showing lesions in the mouth; (F) goat showing haemorrhage around the eyes; (G) goat showing arching of the back; (H) sheep showing signs of diarrhoea (tail soiled with feces).

### Sequencing and phylogenetic analyses

The phylogenetic trees (the maximum likelihood) were constructed based on the obtained sequences of the individual PPRV partial genes (F, N, M, H, L, and P). We selected some sequences representing a high degree of identity to our sequences and representing all lineages to build up our trees. Multiple alignments of these sequences with other sequences in Genebank were made using the Mega-7 package. The phylogeny was conducted using the best-fit model determination, and at least 1,000 bootstraps replicate for the maximum likelihood method, as previously described (Kumar et al., 2014).

### Statistical analysis

We conducted both a descriptive and inferential statistical analysis for our samples. We used Chi-square to test the significance of the difference between frequencies. Descriptive statistical analysis performed to analyses the basic demographics. A *p*-value cut off point of 0.05 at 95% CI used to determine statistical significance. We used the Statistical Packages for Social Sciences [SPSS] version 21.

## Results

### Clinical and necropsy findings

**Figure 3:**
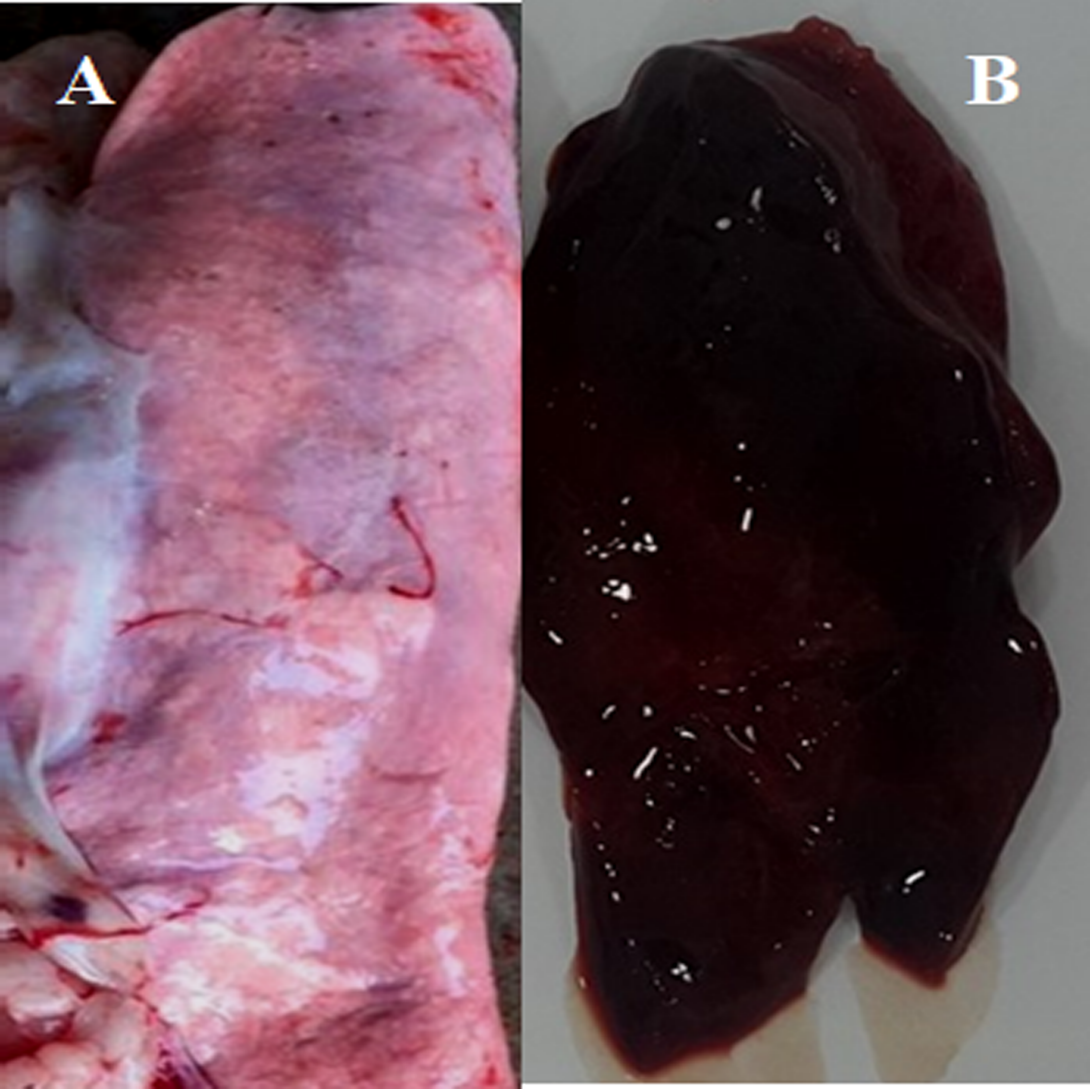
Post-mortem lesions of some normal and PPRV infected sheep. (A) A normal (none-PPRV infected) sheep lung, bright rose in colour; (B) an PPRV-infected lung of sheep showing a dark red colour, congestion and consolidation.

We conducted molecular surveillance of PPRV among small ruminant flocks across the KSA from Jan 2014 to Oct 2016. A total of 223 samples (tissues and swabs) representing 97 suspected PPRV outbreaks were tested by qPCR (Table 1). A KSA map that shows the geographical distribution of the alleged PPRV outbreaks in 10 different regions across the KSA is shown in Fig. 1 (Riyadh, Dammam, Al-Hasa, Hafar Al-Batin, Qasseem, Arar, Tabuk, Madinah, Taif, and Asir). The molecular prevalence of PPRV was mainly based on the testing of 223 samples that represented the 97 outbreaks (Table 1). The observed PPRV outbreaks in small ruminant animals were associated with a wide range of clinical signs. These clinical signs included coughing, sneezing, and nasal discharge (Fig. 2A). Some animals developed erosion and ulceration in and around the corners of the mouth (Fig. 2B). Some animals showed conjunctivitis, excessive tears, lacrimation, corneal opacity, and discharge from one eye. This discharge and conjunctivitis may progress to affect both eyes within 2–3 days of infection onset (Figs. 2C and 2D). Some animals showed ocular infections that progressed to complete blindness one week post-infection (Figs. 2E and 2F). Additionally, some animals showed arching of the back, tails that were soiled with fecal matter, diarrhea, emaciation, and ultimately death at the end of the course of infection (Figs. 2G and 2H). Necropsy examination of some of the PPRV-infected animals revealed congestion and consolidation of the lungs (Fig. 3).

### Real-time and conventional PCR for PPRV detection

We also conducted molecular surveillance to evaluate the PPRV status in small ruminants across the KSA between Jan 2014 and Oct 2016. The same 97-suspected PPRV outbreaks were investigated by RT-PCR. A total of 223 specimens representing 97 outbreaks were tested. Our results show that only 27.8% of the suspected outbreaks were PPRV-positive, with 70% of the tested outbreaks being negative. Meanwhile, 29.1% of sheep and 24% of goats were PPRV genome-positive (Table 1). Meanwhile, 11 of 13 sheep swabs and 8 of 10 goat swabs were positive for the PPRV genome (Table 1). The overall prevalence of PPRV among the tested specimens (tissues and swabs) was 24.8% and 25.8% in sheep and goats, respectively (Table 1).

Six out of the ten regions reported positive PPRV outbreaks (Table 1). The total number of outbreaks in sheep was much higher than that in goat (*p*-value <  0.05). Meanwhile, the prevalence of the PPRV outbreaks was much higher in sheep during 2014 than 2015 in various locations (Table 1). The overall prevalence of the suspected PPRV outbreaks was much higher in small ruminants in the Riyadh region compared to other regions in the Kingdom (Table 1).

### Virus isolation

We used Vero cell lines to propagate and isolate the currently circulating PPRV strains in the KSA. We then selected ten samples from seven of the outbreaks to isolate the PPRV. These samples included swabs and various tissues collected from positive PPRV infected animals. Successful isolation of the PPRV was assessed by the appearance of CPE in the inoculated cells. The presence of the virus was then confirmed by qPCR after three subsequent passages. PPRV-induced CPE in the inoculated Vero cells included rounding of the cells, detachment of the cells from the confluent monolayer sheet, floating of the cells in the cell culture supernatant, and cell death. PPRV isolation was successful for only two out of the ten samples tested. Specimens that did not show CPE were subcultured in parallel for three subsequent passages as well. The first sample was obtained from an RT-PCR-positive sheep lung that was collected from a small ruminant herd in the Riyadh region. The second sample was obtained from a lachrymal swab of a PPRV-positive sheep herd that was also in the Riyadh region (Table 1). The positive lung specimens induced CPE in the three subsequent passages, starting from the first passage. The Ct values for these three passages were 31, 32, and 34, respectively. Furthermore, the positive lachrymal swab induced CPE in the three subsequent passages, with the Ct values of those passages being 32, 34, and 38. We included a negative control (PBS-inoculated) cell culture as well as a positive control (PPRV vaccine Nig-75). The negative control-infected cells did not show any CPE and were RT-PCR-negative in the three subsequent passages. However, the positive control inoculated cells showed CPE in the inoculated cells. The Ct values of the positive control inoculated groups were 34, 33, and 34, respectively (Table 1). The trend of lowering the Ct values with the subsequent passage may be due to the cell death of the virus-infected cells.

### Phylogenetic analysis

A portion of the samples that were positive for the PPRV genome, according to qPCR, was tested by agarose gel electrophoresis. The amplified amplicon size was 448 nucleotides in length. We further selected another PPRV-positive sample showing low Ct value to be sequenced by the NGS method. We tested the collected tissue specimens for the presence of PPRV genomes by qRT-PCR. We used the primers listed above to amplify the partial PPRV-F gene. The size of these amplicons was 448 base-pairs in length. We sequenced several tissue specimens from the suspected outbreaks. We confirmed the identity of the amplified partial PPRV-F gene (448 base pairs) by blasting the sequences against other PPRV isolates in GenBank. All the obtained sequences were identical. We used only one sequence, as represented in our phylogenetic analysis. We analyzed one PPRV-positive sequence per PPRV outbreak in Riyadh. The 448 base-pair sequence of the partial PPRV-F gene originated in PPRV-infected sheep intestine and was deposited in GenBank (accession no: KY798143). This sequence revealed high nucleotide identity to other available PPRV sequences from GenBank, especially to the Ethiopian strain (99%) (accession no: KJ867541.1). We used this Ethiopian strain as a reference sequence for the PPRV genome in our data analysis. Although these specimens were sequenced using NGS to decode the full-length PPRV genome, we were able to obtain only partial PPRV genome sequences from NGS (approximately 45% of the full-length genome of the virus). However, sequencing the PPRV-infected lung tissues from CPE in the Riyadh outbreak by the NGS approach revealed several fragments per gene of the full-length PPRV genome. We assembled a fragment from the PPRV-F gene that spanned 416 nucleotides (accession no: KY679139) from one outbreak in the Riyadh region. The sequence obtained from the partial PPRV-N gene spans a region of 618 nucleotides (accession no: KY679139). Furthermore, another fragment of the M gene that spans 518 nucleotides (accession no: MT421967) was also obtained. This is in addition to the fragments from the PPRV-L, PPRV-P, and PPRV-H genes that span 398, 244, and 229 nucleotides, respectively (received accession numbers: KY684035, KY679140, and KY679136, respectively).

**Table 2:** Summary of SRMV isolation in Vero cell culture.

N.	Region	Sp	Type of sample	Date of Collection	P1			P2			P3		
					CPE	PCR	CT	CPE	PCR	CT	CPE	PCR	CT
1	ASIR	Gt	Lung	30∕12∕2015	-VE	NA	NA	-VE	NA	NA	-VE	NA	NA
2	Ryd	Sh	Lung	24∕1∕2016	-VE	NA	NA	-VE	NA	NA	-VE	NA	NA
3	Ryd	Sh	Lung	29∕3∕2015	+VE	+VE	31	+VE	+VE	32	+VE	+VE	34
			Liver		Toxicity**			NA	NA	NA	NA	NA	NA
4	Ryd	Sh	Lung	6∕1∕2016	-VE	NA	NA	-VE	-VE	-VE	NA	NA	NA
			spleen		-VE	NA	NA	-VE	-VE	-VE	NA	NA	NA
5	Ryd	Sh	Oral swab	22∕5∕2016	-VE	NA	NA	-VE	-VE	-VE	NA	NA	NA
			Lachrymalswab		+VE	+VE	32	+VE	+VE	34	+VE	+VE	38
6	Ryd	Gt	Oral swab	23∕5∕2016	-VE	NA	NA	-VE	-VE	-VE	NA	NA	NA
7	Ryd	Sh	Lachrymalswab	1∕6∕2016	-VE	NA	NA	-VE	-VE	-VE	NA	NA	NA
8	(-Ve) Cl	NA	PBS	NA	-VE	NA	0	-VE	NA	0	-VE	NA	0
9	(+Ve) Cl	NA	Vaccine*	NA	+VE	+VE	34	+VE	+VE	33	+VE	+VE	34

**Notes.**

Ryd Riyadh sh sheep Gt goat P1-P3 passages 1, 2 and 3 (+Ve) positive (-Ve) negative CPE cytopathic effect CT cycle threshold Cl control

**Figure 4:**
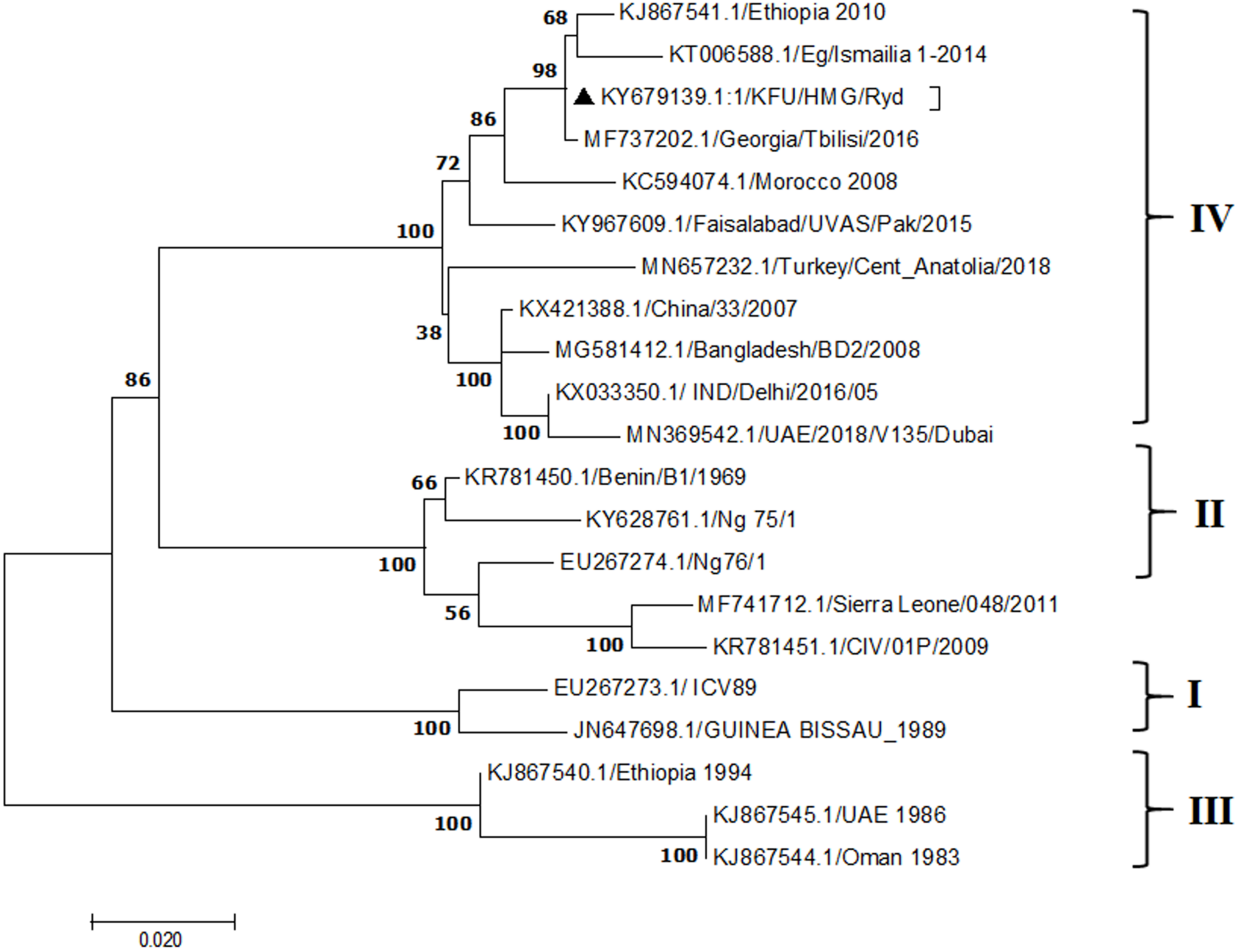
Phylogenetic analysis of PPRV isolated from small ruminants in the KSA based on the partial sequences of the N gene. The maximum likelihood of the decoded PPRV sequences isolated from lung tissues from the Riyadh outbreak based on the partial PPRV-N gene. The PPRV-Saudi isolates are indicated by a black triangle.

**Figure 5:**
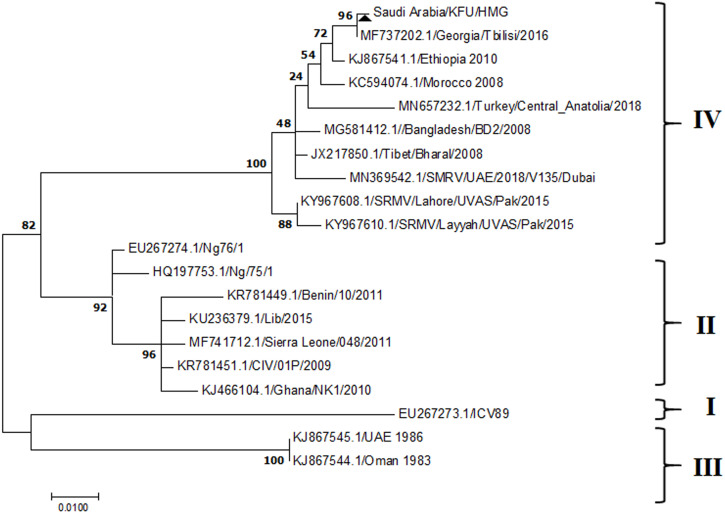
Phylogenetic analysis of PPRV isolated from small ruminants in the KSA, based on the partial sequences of the F gene. The maximum likelihood of the decoded PPRV sequences isolated from lung tissues from the Riyadh outbreak, based on the partial PPRV-F gene. The PPRV-Saudi isolates are indicated by a black triangle.

**Figure 6:**
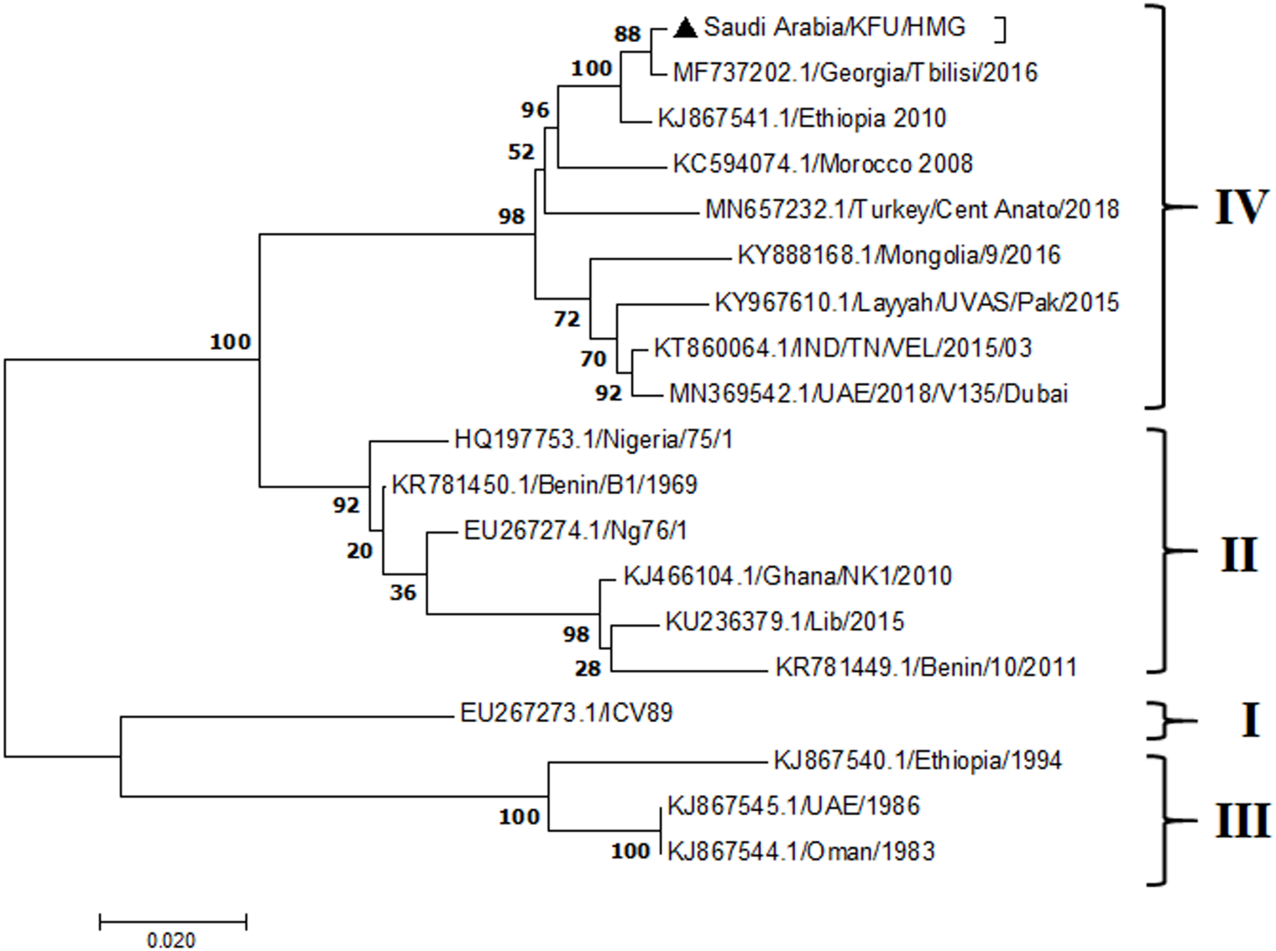
M Gene. The maximum likelihood of the decoded PPRV sequences isolated from lung tissues from the Riyadh outbreak, based on the partial PPRV-M gene. The PPRV-Saudi isolates are indicated by a black triangle.

**Figure 7:**
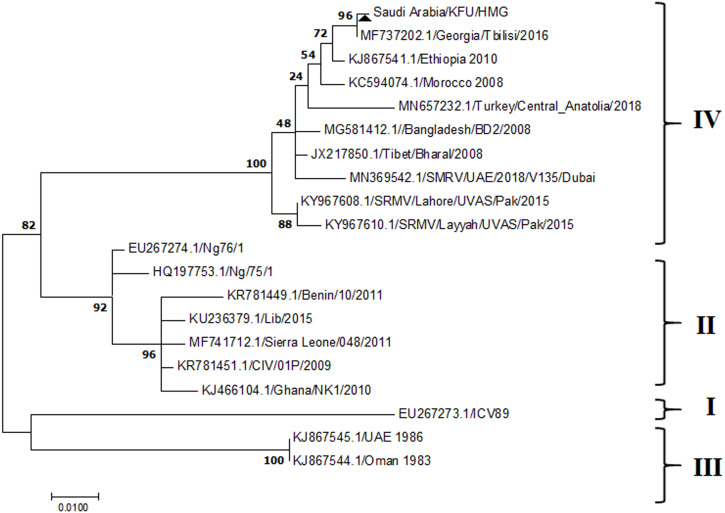
L Gene. The maximum likelihood of the decoded PPRV sequences isolated from lung tissues from the Riyadh outbreak, based on the partial PPRV-L gene. The PPRV-Saudi isolates are indicated by a black triangle.

**Figure 8:**
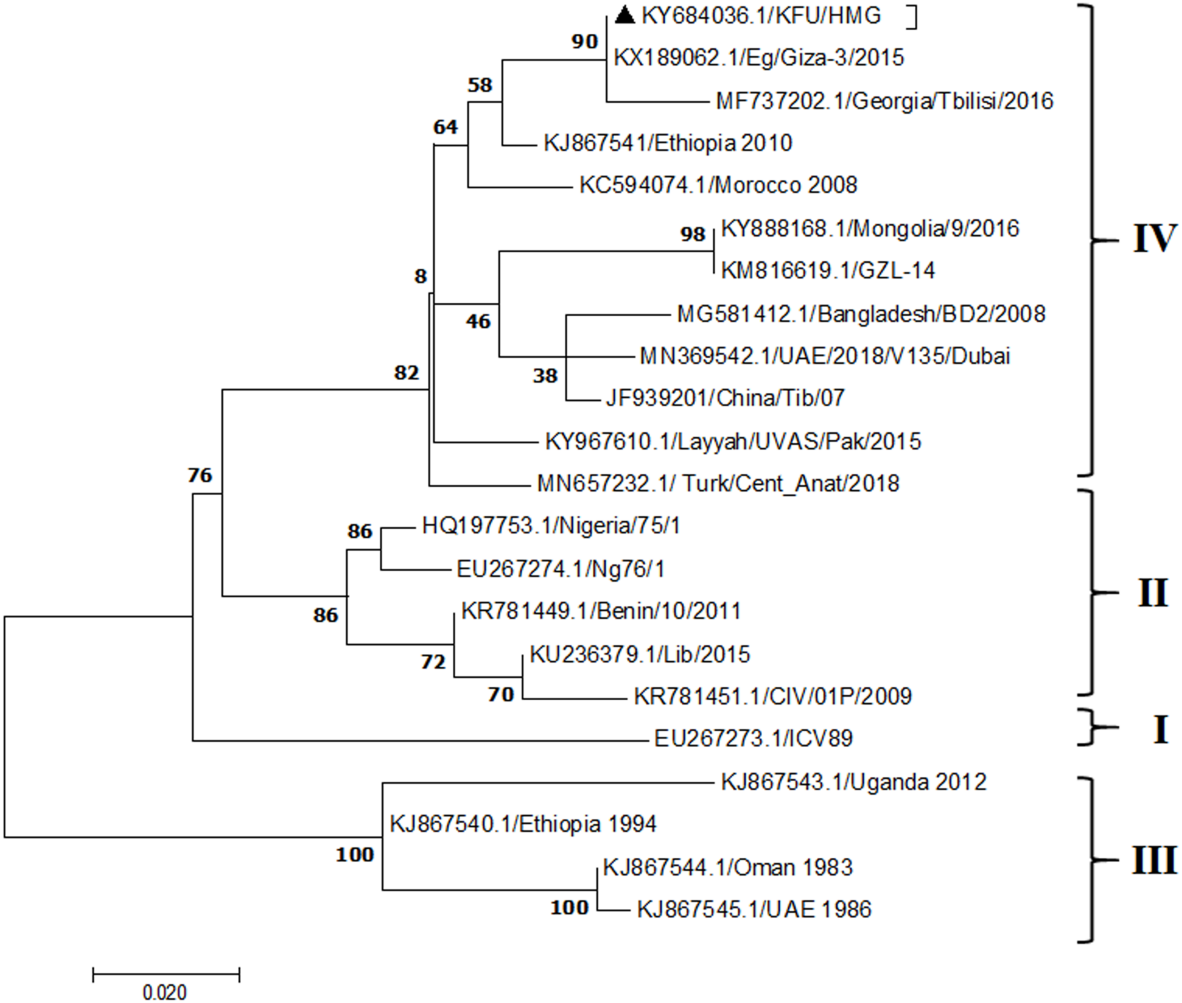
Phylogenetic analysis of PPRV isolated from small ruminants in the KSA, based on H gene. The maximum likelihood of the decoded PPRV sequences isolated from lung tissues from the Riyadh outbreak, based on the partial PPRV-H gene. The PPRV-Saudi isolates are indicated by a black triangle.

**Figure 9:**
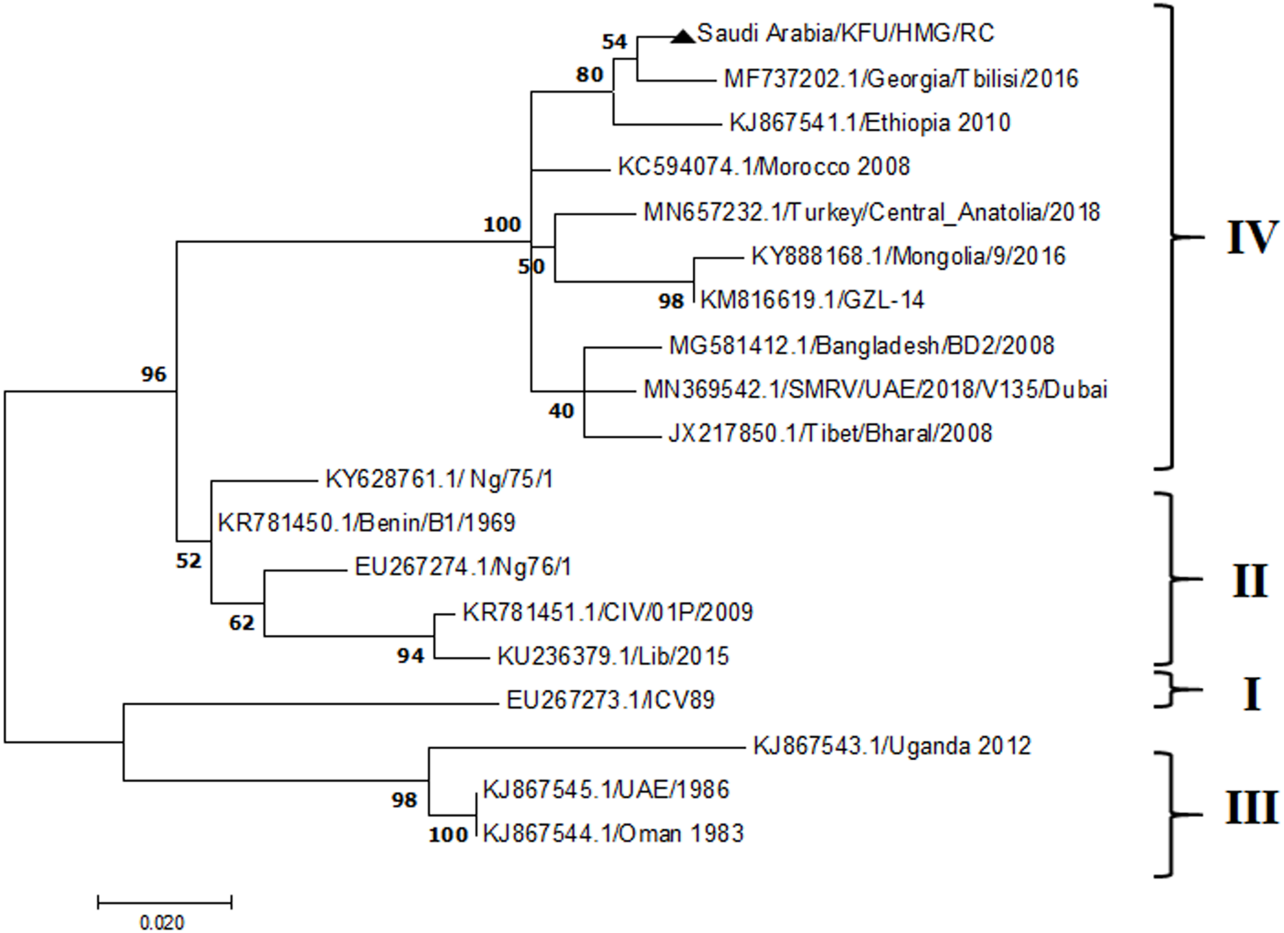
P Gene. The maximum likelihood of the decoded PPRV sequences isolated from lung tissues from the Riyadh outbreak, based on the partial PPRV-P gene. The PPRV-Saudi isolates are indicated by a black triangle.

## Discussion

The PPRV infected small ruminants are usually showing signs of stomatitis and pneumonitis in most of the cases (Chauhan et al., 2009). These observations are very much consistent with our study (Fig. 2). Despite the absence of the full-length genome sequences of the local Saudi isolates of the PPRV, some studies previously identified important key fragments of the N gene of the virus (Ahmed et al., 2018). To address this question, we conducted molecular PPRV surveillance across the KSA from Jan 2014 to Oct 2016. We tested 223 specimens from different small ruminant flocks that represented 97 outbreaks across the country by qPCR. We did our best to collect at least 10% of each investigated outbreak; however, this was hampered in some cases by the consonant from the owners of each herd.Almost 50% (*n* = 49) of the tested outbreaks were from animals in the Riyadh region. The high prevalence of PPRV among small ruminants in the Riyadh region may be attributed to the high population density of small ruminants in this region. Furthermore, several public small ruminant markets are located around this area. These markets receive animals externally from the Jeddah Islamic Port and internally from different regions of the country. This is in addition to the active movement of small ruminants between Riyadh and other regions across the country. Of the 49-suspected PPRV outbreaks recorded in Riyadh, 15 outbreaks (ten sheep and five goats) were positive for PPRV (30.6%) (Table 1). Meanwhile, five out of the 20-suspected PPRV outbreaks tested in Asir (Southern region) were positive for PPRV. The high prevalence of PPRV among animals in the southern region may be related to the shared border with Yemen, which is a PPRV endemic country (Dhar et al., 2002). We selected ten- samples, including tissues from lungs, liver, and spleen, as well as oral and lachrymal swabs for virus isolation. The rationale underlying the use of different types of specimens for the viral isolation was to identify the best type of specimen for the isolation of the Saudi isolates of PPRV. The main purpose of PPRV isolation was to prepare a virus stock, which we may use for further molecular characterization of the circulating strains of PPRV in Saudi Arabia. We may use these isolates in the preparation of some locally prepared vaccines in the future. PPRV can be isolated using Vero cell lines (Meng, Dou & Cai, 2014). The CPE produced by PPRV infection in the Vero cell lines included cell rounding and clustering together, detachment of the cells from the monolayer, and cell death within four days of infection (Hegde et al, 2009). Several attempts made to isolate PPRV from the specimens collected from outbreaks of PPRV that confirmed by using the Vero cell lines. Ten qPCR -positive samples, including six tissue specimens were collected (four lungs, one liver, and one spleen) and four swabs (two oral and two lachrymal) (Table 2). One liver tissue specimen from an outbreak in sheep from the Riyadh region showed signs of cell toxicity in the Vero cells. This specimen was excluded from the isolation process without any further testing. Isolation of PPRV was only successful in two specimens, one from lung tissues and the other from a lachrymal swab collected from a sheep (Table 2). Isolation success parameters mainly based on the presence of CPE and the detection of PPRV in tissue culture supernatants by qPCR in three subsequent passages (Table 2). The increase of the Ct values of some with the subsequent passages in some specimens may be attributable to the low viral loads, degradation of viral RNA, as well as the presence of some toxins in the original specimens, which may lead to early cell death. Furthermore, this could be the reason why, in this study, it was not possible to obtain the full-length genome sequence of the PPRV from the original specimens, as discussed below. Our study is suggesting that PPRV isolation is not a reliable diagnostic method for the detection of PPRV compared with qPCR assays (Balamurugan et al., 2010). We planned to decode the full-length genome sequences of these PPRV isolates. Several attempts have been made to achieve this goal in our laboratory and in consultation with some commercial companies. However, due to some reasons such as low viral load in the specimens, RNA degradation, and the presence of certain nonspecific inhibitors, we were only able to report approximately 50% of the PPRV genome. We mapped the obtained gene fragments, which span different structural genes and deposited this information in Genebank. It is now well established that the PPRV-N gene sequences can be used to identify the PPRV lineage circulating in a certain region (Munir, Zohari & Berg, 2013). Phylogenetic analysis based on the obtained PPRV-N gene revealed that our isolate from Riyadh clustered with other members of PPRV lineage IV (Fig. 4). They are also closely related to isolates from Georgia Tbilisi (MF737202.1) and isolates from Egypt (KT006588.1) (Fig. 4). This result is consistent with a previous report on PPRV-N gene sequences that were recorded in 1999 (accession no. DQ840195.1, DQ840197.1, and DQ840196.1). However, these sequences did not fall in the same regions that our sequence did. Thus, we did not include them in our phylogenetic trees. Furthermore, the phylogenetic tree based on the partial PPRV-F gene from two far regions in the country located in Riyadh (accession number FR667645.1) and Hafar Al-Batin (FN995267.1) showed that these two isolates were closely related to the other PPRV-F gene sequences reported between 1994 and 2004 (Data not shown). This outcome is consistent with the previous reports of PPRV sequences from the KSA (accession no: FR667645.1 and FN995267.1) published in 1994 and 2004, respectively. These two PPRV isolates were classified as being in lineage IV (Kumar et al., 2014). Results obtained from the phylogenetic trees based on the PPRV (PPRV-M, L, H, and P) genes in this study are consistent with those of the PPRV-N (Fig. 4) and PPRV-F gene trees(Fig. 5). This result confirms that the reported strains from this study from the KSA belong to lineage IV (Figs. 6–9). Typically, both the partial PPRV-F and -N genes were the candidates of choice for the identification of PPRV lineages (Kumar et al., 2014; Kumar et al., 2013; Woma et al., 2016). However, one new study reported the possibility of using the circulation of two distinct PPRV lineages in small ruminants in Nigeria (Lineage II and IV) (Baron & Barrett, 1995; Naveen et al., 2014; Woma et al., 2016). Our PPRV-F gene sequences suggest that there may be some genetic variation between the PPRV isolates that were reported between 1994 and 2004 and the isolate strains used in this study. Another possibility is the potential circulation of more than one lineage of PPRV among small ruminant flocks in the KSA. Interestingly, our isolates are clustered together with the recently reported PPRV from Georgia (Fig. 5). Additionally, Fig. 8 is showing the PPRV Saudi strains in the current study were closely related to other strains from Egypt based on the H gene. This may highlight the potential roles of the trade and importation of small ruminants from different parts of the world, such as Georgia Tbilisi, Egypt, and Ethiopia, in the transmission of PPRV (Donduashvili et al., 2018). In UAE and Oman, lineage III reported in small ruminants in 1986 (accession no: KJ867545.1 and KJ867545.1, respectively). Recently, another isolate belongs to lineage IV recently reported from UAE deposited in the GenBank in 2018, which showed a 95.94% identity to our N gene sequences (accession no: MN369542). Interestingly, lineage III never reported in KSA, yet despite the KSA, UAE, and Oman share the borders in addition to some border crossing of small ruminants during the grazing and trading. Further studies are needed to do molecular characterization of the circulating strains of PPRV in the Arabian Peninsula. We believe that this study will have a strong impact on the global PPRV eradication campaigns, including those in the KSA.

## Conclusions

Our data demonstrate that PPRV lineage IV was still circulating and causing economic problems in the small ruminants industry in the KSA between 2014 and 2016. Continuous monitoring and surveillance of the situation of PPRV not only in the Arabian Peninsula but also worldwide is highly recommended. Identification of the endemic PPRV regions across the KSA may contribute significantly to fine-tuning the PPRV vaccination programs and, subsequently, to the control and global eradication campaigns against PPRV.

##  Supplemental Information

10.7717/peerj.9035/supp-1 Figure S1 Summary of real time PCR testing of PPRV infected VERO cells

10.7717/peerj.9035/supp-2 Supplemental Information 1 Raw data PPRV isolation via Vero cell line

## Additional Information and Declarations

### DNA Deposition

The following information was supplied regarding the deposition of DNA sequences: Data is available at GenBank: KY798143, KY679139, KY679139, MT421967, KY684035, KY679140 and KY679136.

### Data Availability

The following information was supplied regarding data availability: Raw data are available as a Supplementary File.
