# Supplementary material for: Prevalence and molecular characterization of some circulating strains of the peste-des-petits-ruminants virus in Saudi Arabia between 2014–2016
Source: PeerJ. 2020 May 27;8:e9035. doi: 10.7717/peerj.9035 (PMC7261128; doi:10.7717/peerj.9035)
Supplement: Figure S1 [file peerj-08-9035-s001.docx]

**Numeric data for Table 2**

**Supplementary Figure 1**. Summary of real time PCR testing of PPRV infected VERO cells

| **Passage** | **Tissue** | | | **Swabs** | | |
| --- | --- | --- | --- | --- | --- | --- |
|  | **No tested** | **+Ve** | **%** | **No tested** | **+Ve** | **%** |
| **P1** | 6 | 1 | 16.6 | 4 | 1 | 25 |
| **P2** | 6 | 3 | 50 | 4 | 2 | 50 |
| **P3** | 6 | 3 | 50 | 4 | 2 | 50 |

P1-P3 (PPRV cell culture passages 1-3), +Ve (No of positive real time PCR), % (percentage of positive real time PCR out of the total number of tested samples)
